# The Relationship between Gastroduodenal Pathologies and *Helicobacter pylori* *cagL* (Cytotoxin-Associated Gene L) Polymorphism

**DOI:** 10.5152/tjg.2023.22274

**Published:** 2023-04-01

**Authors:** Doğukan Özbey, Süleyman Demiryas, Seher Akkuş, Nuray Kepil, Harika Öykü Dinç, Nesrin Gareayaghi, Mehmet Demirci, Enes Ali Kurt, Ömer Uysal, Suat Sarıbaş, Hrisi Bahar Tokman, Bekir Kocazeybek

**Affiliations:** 1 Department of Medical Microbiology, İstanbul University Cerrahpaşa Faculty of Medicine, İstanbul, Turkey; 2 Department of General Surgery, İstanbul University Cerrahpaşa Faculty of Medicine, İstanbul, Turkey; 3 Department of Medical Pathology, İstanbul University Cerrahpaşa Faculty of Medicine, İstanbul, Turkey; 4 Department of Pharmaceutical Microbiology, Bezmialem Vakıf University Faculty of Pharmacy, İstanbul, Turkey; 5 University of Health Sciences, İstanbul Hamidiye Etfal Training and Research Hospital, İstanbul, Turkey; 6 Department of Medical Microbiology, Kırklareli University Faculty of Medicine, Kırklareli, Turkey; 7 Department of Gastroenterology, İstanbul University Cerrahpaşa Faculty of Medicine, Istanbul, Turkey; 8 Department of Biostatistics, İstanbul University Cerrahpaşa Faculty of Medicine, İstanbul, Turkey

**Keywords:** CagL, duodenal ulcer, gastric cancer, helicobacter pylori, polymorphism, vacA

## Abstract

**Background:**

The polymorphisms in the region between 58 and 62 amino acids of the 194-amino acid CagL protein (CagL hypervariable motif) affect the binding affinity of CagL to integrin α5β1 (ITGA5B1) receptor in host epithelial cells and have an effect on the development of various gastrointestinal diseases. We aimed to evaluate the associations of gastroduodenal pathologies, with the polymorphisms of *cagL* gene of *Helicobacter pylori* (H. pylori) and also associations between *vacA* genotypes and *cagL* polymorphisms.

**Methods:**

A total of 19 gastric cancer, 16 duodenal ulcer, and 26 non-ulcer dyspepsia patients were included in this case-control study. All cases had *H. pylori*. A fragment of 651 bp from gene *cagL* (hp0539) and *cagA*, *vacA* genes was amplified by polymerase chain reaction. Purified polymerase chain reaction products were sequenced by Sanger sequencing, and nucleotide sequences were translated into amino acid sequences.

**Results:**

All of the *H. pylori *strains had *cagL* and *cagA* genes. In the 16 (84%) gastric cancer cases, the D58 amino acid polymorphism was significant than the 4 (15.4%) duodenal ulcer cases (*P = .*029), and the D58/K59 amino acid polymorphism was significant in 12 (63.1%) of the gastric cancer cases than 1 (3.85%) duodenal ulcer case (*P = .*008). D58/K59 and DKIGQ (n = 10; 52.63%) were the most common polymorphisms in the gastric cancer and were associated with the *vacA *genotype s1/m2, respectively (*P = .*022 and *P = .*008). The D58/K59 amino acid polymorphism was found to have a significant Odds Ratio (OR) value of 8.9 (*P = .*0017) in multivariate logistic regression analysis.

**Conclusions:**

The risk of gastric cancer development is 8.9 times higher with D58/K59 polymorphism.

**Main Points**

- According to The International Agency for Research on Cancer (IARC), gastric cancer is the third leading cause of death from cancer.
- Helicobacter pylori, which is also classified as a Class I carcinogen according to the same institution, has been accepted as a bacterium directly related to gastroduodenal pathologies in the stomach, especially gastric cancer.
- The CagL protein possessed by this bacterium directly affects the adhesion of the bacterium to the host gastric epithelial cells and the entry of the main oncoprotein, CagA, into the cell.
- Certain polymorphisms, such as the D58/K59 polymorphism in the amino acid sequence of the CagL protein, may contribute to this effect.

## Introduction

*Helicobacter pylori *(*H. pylori*) is one of the most common causes of cancer-related deaths^1^ and is thought to infect half of the world’s population.^2,3^ *H. pylori* can contribute to the development of various gastrointestinal diseases.^4-6^ Cytotoxin‐associated gene L protein (*CagL*) is a virulence factor used for attachment to the epithelial cells and encoded by the 165-bp gene region in the CagPAI region. *CagL* is located in the pilus of the Type 4 Secretion System and attaches to the integrin α5β1 (ITGA5B1) receptor in epithelial cells. The Arginine-Glycine-Aspartate (RGD) motif, the adjacent helper sequence (RHS), and Phenylalanine-Glutamate-Alanine-Asparagine-Glutamate (FEANE) motif mediate the binding of *CagL*.^1,7-13^

It has been suggested that the polymorphisms in the region between the 58 and 62 amino acids of the CagL protein, which is called the CagL hypervariable motif (CagLHM), affect the binding affinity of CagL to ITGA5B1 and may have an effect on the development of various gastrointestinal diseases.^1,7-12^ CagL has 6 chains numbered from α1 to α6 in its crystal structure, and the CagLHM region is located in the hinge region between the *α1* and *α2* chains.^1,12^ The CagL protein is thought to be a good vaccine target because of its surface expression and the presence of various motifs.^14,15^

CagA, another virulence factor of *H. Pylori*, undergoes tyrosine phosphorylation after entering the host cell.^16,17^ The fourth amino acid (tyrosine) of the EPIYA motif, which is located at the C-terminal is the main source of this phosphorylation.^16^ After binding of *H. pylori* to the host epithelium via CagL, CagA is translocated into the cell, which has the main oncogenic effect.^17^ Various amino acid polymorphisms between amino acids 58 and 62 in the CagLHM region might contribute to the development of different gastrointestinal pathologies.^7-13^ The VacA toxin of *H. pylori* is a toxin synthesized to form selective membrane channels.^18^ The *vacA* gene is divided into groups with different alleles according to the signal region (*s*) and middle (*m*) at the amino terminus. It can contain one of the *s1a, s1b, s1c*, and *s2* alleles in the s region and one of the *m1*, *m2a*, and *m2b* alleles in the m region.^18-20^

We aimed to evaluate the associations of gastroduodenal pathologies with the amino acid polymorphisms of CagL protein detected in *H. pylori* DNAs isolated from patients. We also discuss whether our findings might be specific to the Turkish setting and investigate the relationship between the *cagL* polymorphisms and *vacA* genotypes.

## Materials and Methods

### Study Design and Patients

This case-control study was conducted between 2019 and 2020. The 2 patient groups, comprising a total of 61 patients (19 gastric cancer [GC] and 16 duodenal ulcer [DU] patients; mean age, 57.316 years for GC and 42.5 years for DU patients), and a control group, comprising a total of 26 individuals with non-ulcer dyspepsia (NUD) (mean age, 51.77 years; age range 22–76 years), were enrolled. All subjects had *H. pylori*. The control group was matched with the patient group (*P* > .05). The antrum and corpus biopsies were used for molecular studies. We excluded patients who were younger than 18 years old, had previous gastric surgery or *H. pylori *eradication treatment, or had a history of therapy with antibiotics, antisecretory drugs, bismuth salts, or sucralfate in the month prior to sampling.

Collected biopsies were transferred immediately in Brucella broth to the laboratory. The study was approved by the Clinical Research Ethics Board of Istanbul University Cerrahpasa Faculty of Medicine (Ethical approval No: A-08/2019) and recognized the standards of the Declaration of Helsinki. All patients gave informed consent to participate in the study.

### Molecular Methods

#### *ureC* Gene Detection in *H. pylori*

The presence of *H. pylori* was determined histopathologically from biopsy samples. DNA isolation was done using the QIAamp DNA Mini Kit (Qiagen GmbH, Hilden, Germany). In order to verify *H. pylori* DNA, the *ureC* gene region (*glmM*) of *H. pylori* was determined by the qPCR method using a Fluorion device (Iontek, Istanbul, Turkey) and *H. pylori*-QLS 1.0 kit (Iontek, Istanbul, Turkey) device.

#### Amplification of the *H. pylori cagA* and *vacA* Genes

The *cagA*, *vacAs1/s2*, and *vacAm1/m2* genotypes were determined using a molecular PCR technique using specific primers. All primer sets used were selected from the published studies and are shown in Table 1.^7,10,20,21^ The study protocol was as follows: initial denaturation at 95°C for 2 minutes, followed by 45 cycles of 95°C for 30 seconds, 45 seconds at 53°C, and 45 seconds at 72°C. The final elongation was performed for 5 minutes at 72°C.

#### *CagL* Gene Amplification by Polymerase Chain Reaction

A fragment of 651 bp from gene *cagL *(*hp0539*) gene was amplified by PCR using primers *cagL* sense and *cagL* antisense. The reaction mixture had a final volume of 25 μL and contained 1 μL dimethyl sulfoxide (DMSO), 4.2 μL PCR buffer, 0.7μL of each oligonucleotide, 0.3 μL of Taq recombinant DNA polymerase (Invitrogen, Massachusetts, USA), 1.5 μL of DNA, and 16.6 μL distilled water. The conditions used were 1 cycle at 94°C for 5 minutes; 45 cycles at 94°C for 30 seconds, 55°C for 30 seconds, and 72°C for 1 minute; and 1 cycle at 72°C for 7 minutes. Each reaction included a positive (DNA from strain 26695) and a negative control. All reactions were performed in a Mastercycler Ep gradient thermocycler (Eppendorf, Hamburg, Germany). Polymerase chain reaction products were analyzed by agarose gel electrophoresis at 1.5% and stained with ethidium bromide. A second PCR was performed with DNA from those strains that were negative in the first reaction, using primers *cagL*Fwd-2 and *cagL*-16, which amplified a 165-bp product.

#### Purification and Sequencing of Polymerase Chain Reaction Products

Polymerase chain reaction products were cleaned with ExoSAP. For the purification of *H. pylori*/*cagL* gene, positive PCR products from the first and second PCR stages, 4 μL of PCR product, and 1 μL of Exo-Sap (Exonuclease 1 and Shrimp Alkaline Phosphatase enzymes) were mixed to remove unbound DNA and primers from the reaction tube. Exonuclease 1 enzyme was used to digest unbound primers in the medium, and the Shrimp Alkaline Phosphatase enzyme was used to digest unbound dNTPs in the medium. The Exo-Sap program of the PCR device was selected. Tubes were placed in the instrument, the instrument was turned on, and a PCR of 30 minutes was performed.

Purified PCR products were sequenced by the Sanger Sequencing method on ABI 3730XL (Applied Biosystems, Foster City, California, USA) using the Big Dye™ Terminator v3.1 Cycle Sequencing Kit (Thermo Fisher, Massachusetts, USA) kit. The nucleotide sequences obtained were aligned using MEGA 7.0 (Mega software, USA) and translated into amino acid sequences using ExPASy (Swiss Biotechnology Institute, Lausanne, Switzerland).

Evolutionary analysis was performed using the neighbor-joining method.^22^ The length of the branches of the optimal tree was determined as = 0.32397031. The evolutionary distance was generated by the software using the Poisson correction method.^23^ The analysis included 48 amino acid sequences. All positions with gaps and missing data have been eliminated. There were a total of 194 positions in the final data set. All evolutionary analyses were performed using MEGA7 software.^24^

#### Determination of *cagL* Gene Polymorphisms

The amino acid sequences obtained were aligned in the MEGA 7.0 program and compared with the *H. pylori* 26695 references strain for the determination of the amino acid polymorphisms of each DNA sample. The *H. pylori* 26695 (ATCC 700392; NCBI:txid85962) reference strain was used because it is a well-known strain with full genome and virulence properties.

### Statistical Analyses

The Statistical Package for the Social Sciences version 25.0 (IBM Corporation, Armonk, NY, USA) program was used. Fisher’s exact test was used for comparisons of CagL amino acid polymorphisms in the groups, and the results were shown as Benjamini-Hochberg corrected *P*-values. In the context of determining the cause and effect relationship with the explanatory variables, single amino acid polymorphisms (D58, K59, I60, S61, Q62) and amino acid polymorphic combinations (D58/K59, D58/E59, N58/K59, D58/K59/I60, D58/K59/Q62, D58/K59/I60/Q62, and D58/K59/I60/G61/Q62), along with other group variables (<55 years and 55 years, female/male), were included in a binary logistic regression (multivariate logistic regression) analysis. Variables were analyzed at a 95% confidence level and *P* <.05 was considered significant.

## Results

The mean ± SD (min–max) age of GC, DU, and NUD cases were 57.316 ± 12.93 (31–79), 42.5 ± 10.97 (19–63), and 51.77 ± 15.93 (22–76), respectively. All *H. pylori* strains had *cagA *and *cagL *genes. The baseline characteristics are shown in Table 2.

**Figure 1.**
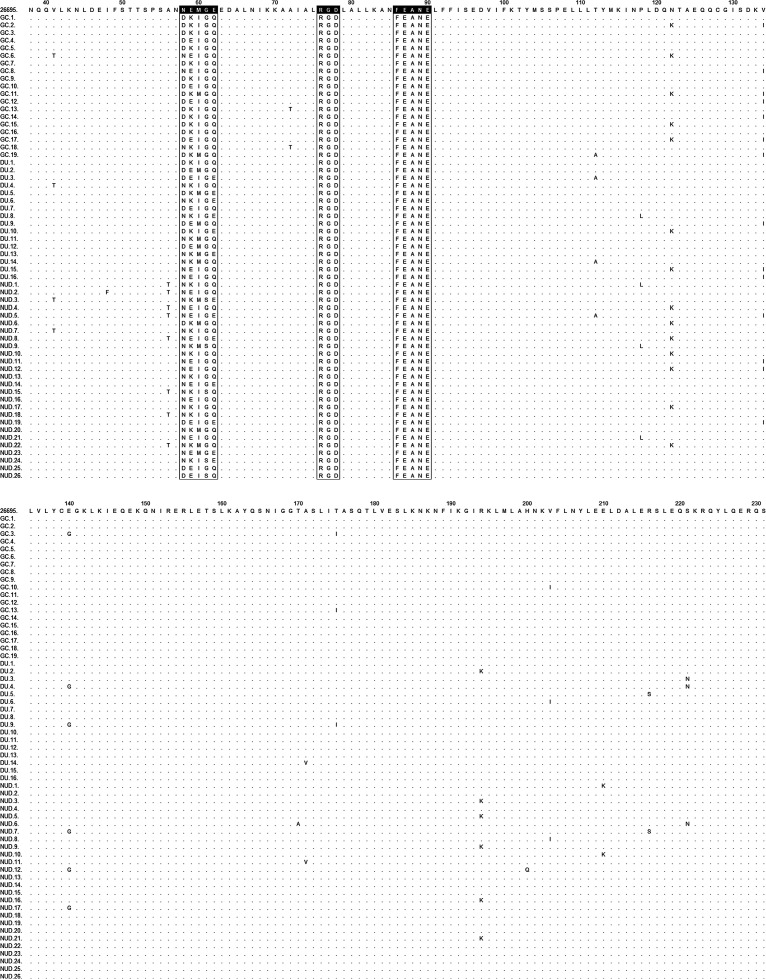
The sequences of 61 CagL (+) *H. pylori* DNA isolates that were aligned with the sequence of strain ATCC 26695.

**Figure 2.**
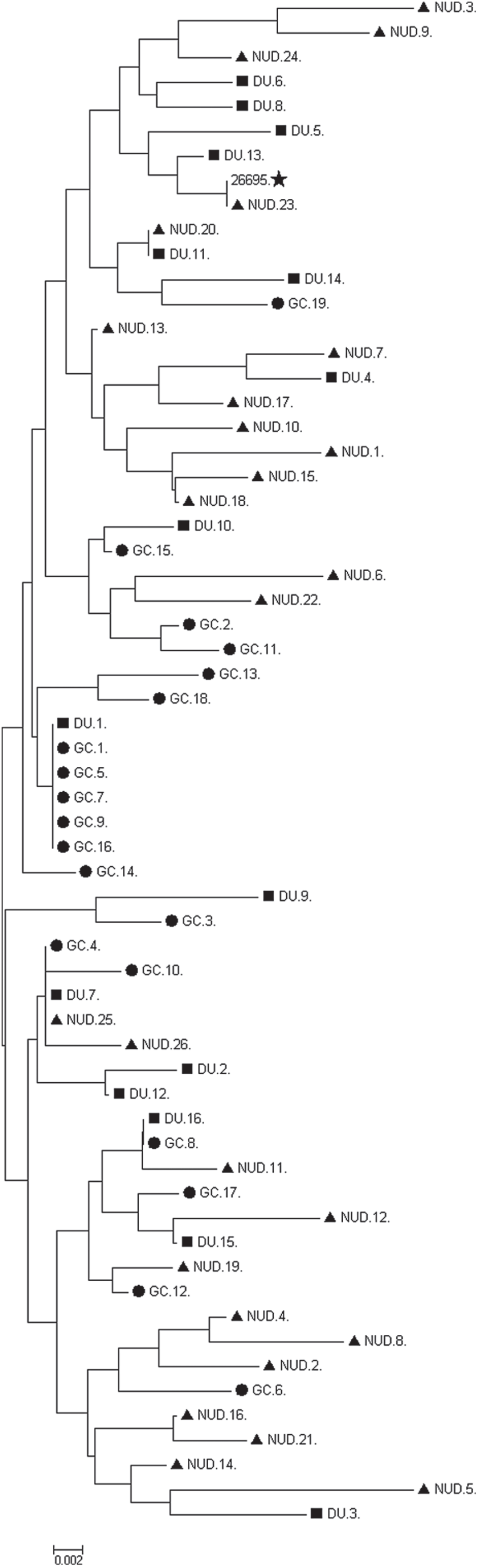
The phylogenetic tree prepared on the basis of amino acid polymorphisms detected in the study and control groups.

The sequences of 61 *cagL* (+) *H. pylori* DNA isolates were aligned with the sequence of strain *H. pylori* 26695 (ATCC 700392) (Figure 1). The Arginine-Glycine-Aspartate (RGD) and RGD Helper Sequence (RHD) motifs are identical in all strains. The phylogenetic tree prepared on the basis of amino acid polymorphisms is shown in Figure 1. All of the 61 amino acid sequences are shown in Figure 2.

Table 3 shows the comparisons of the study and control cases for single amino acid polymorphisms. The D58 polymorphism was detected in 16 (84%) and 4 (15.4%) of GC and NUD cases, respectively (OR: 29, *P = .*0001). The D58 polymorphism was detected in 8 DU (50%) and 4 NUD (15.4%) cases (OR: 5.5, *P = .*032). In 24 (68.6%) of the GC + DU cases, the D58 polymorphism was significant (OR: 12, *P = .*0001) when compared to the NUD cases.

Table 4 shows the comparison of the study and control cases in terms of the 58 and 59 amino acid polymorphisms. The D58/K59 polymorphism was detected in 12 (63.1%) of the GC cases and in 1 (3.85%) NUD case. A significant difference was found between the groups (OR: 42, *P = .*0001), and there was a significant difference between 15 (42.9%) GC + DU cases and 1 (3.8%) NUD case (OR: 18.75, *P = .*001).

The DKIGQ polymorphism was detected in 10 (52.63%) of the GC cases but not detected in the NUD cases (OR: 58.5, *P = .*0065). When comparing the GC + DU and NUD cases, the DKIGQ polymorphism was detected in 11 (31.43%) of the GC + DU cases and in none in the NUD group (OR: 24.8, *P = .*0290).

The D58/K59/Q62 and D58/K59/I60 polymorphisms were detected in 12 (63%) and 10 (52.6%) of the GC and NUD cases (OR: 58), respectively. Association between *cagL* polymorphisms and *vacA* genotypes are shown in Table 5a and 5b. D58/K59 and DKIGQ were the most common polymorphisms in the GC cases and were associated with the *vacA* genotype s1/m2, respectively (*P* = .022 and .008) (Table 5a–5b).

D58, D58/K59, N58/K59, D58/K59/I60, D58/K59/Q62, D58/E59/I60/Q62, DKIGQ polymorphisms, with age group variables (<55 years and 55 years) and gender parameters were included as independent variables in the GC cases in the logistic regression analysis. The D58/K59 polymorphism was found to have a significant OR value of 8.9 (*P = .*000366) (Table 6). In cases with DU, the D58 polymorphism, age group (< 55 years and 55 years), and gender were included and the D58 polymorphism was excluded during the analysis steps. Only <55 age variable had an OR significantly lower than 1 (*P = .*0075) (Table 7). The independent variables that we identified in the univariate analysis in GC + DU cases were D58, D58/K59, D58/K59/Q62, and D58/K59/I60/Q62 and DKIGQ polymorphisms with age and gender were included and only D58/K59 was determined as highly significant (OR: 6.5, *P = .*0017) (Table 8).

The most detected polymorphism outside of the CagLHM region was I134 and followed by K122, I175 and T72 T41, A112, G140, and I203 polymorphisms. The most detected I134 polymorphism in the GC cases was also detected in 4/26 (15.38%) NUD cases but not significant (*P = .*09). All polymorphisms detected outside of were shown in Supplementary Material 1 and in Figure 2.

## Discussion

It is of utmost importance for public health to catch the development of GC at the earliest pre-atrophy stages and to direct the treatment strategy accordingly. It has been proposed that various polymorphisms in the 58-62 amino acids in the *cagL* region are effective in the development of various gastrointestinal pathologies.^1,7-12,25^

The D58 polymorphism was significantly higher in our GC (OR: 29), DU (OR: 5.5), and GC + DU cases (OR: 12). This polymorphism lost its significance in the multivariate analysis. The significantly increased D58/K59 polymorphism was also detected in the GC cases. In the study by Yadegar et al.^8^ the N58, I60, and Q62 polymorphisms were detected most frequently in GC cases, and in Cherati et al’s^1^ study, the most common polymorphisms were E59 and Q62 in their GC cases. Ogawa et al^25^ reported that the K59 polymorphism was significantly higher in GC cases. Shukla et al^9^ found the D58 polymorphism more frequently in the GC cases (61.1%). Yeh et al^10^ reported that the E59 polymorphism was most frequent in the GC cases (68.2%). Román-Román et al^7^ reported that the K59 polymorphism was found in 94% of chronic gastritis cases. Gorrell et al^26^ reported that the N58, E59, I60, and Q62 polymorphisms correlated with the development of GC, but D58, K59, M60, and E62 were negatively correlated in a review. Our data on the frequency of Q62 was similar depending on the geographic proximity of the Iranian Yadegar et al’s^8^ and Cherati et al’s^1^ studies.

D58 polymorphism stands out significantly in our GC (OR:29) cases compared to the control cases. The variations of CagLHM polymorphisms in the world can be explained by the regional geographical differences. Gorrell et al^26^ suggested that this significant geographic diversity was observed in the CagLHM sequences, which might have occurred specifically as a result of the co-evolution of *H. pylori* and the host. In addition, while the same researchers draw attention to the strong positive correlation between the I60 and E59 polymorphisms and GC, on the other hand, they emphasized that the D58 and K59 polymorphisms are detected at a high rate in GC cases, and this might be more related to the diversity of *H. pylori* circulating locally and their adaptation to the host.

The Q62 polymorphism was high (100%) in our GC cases, with the I60 polymorphism ranked second (89%). In our univariate analysis, D58 (84.2%) was found to be significantly associated with GC cases (OR: 29) but was not significant in logistic regression analysis. The D58/K59 polymorphism was the most common, occurring in 12 (63.16%) of our GC cases, while the frequency of the same combination was 15 (42.9%) in our GC + DU cases. In the intergroup comparison, the presence of the D58/K59 polymorphism was significantly higher in GC cases than in NUD cases (OR: 42). In the multivariate analysis, the D58/K59 polymorphism was determined as an independent risk factor (OR:8.9), which significantly increased the risk of gastric carcinogenesis. The D58/K59 polymorphism was significantly higher in GC + DU cases (OR: 18). Shukla et al^9^ reported that the D58/K59 polymorphism increased the GC risk by 2.8 times. Yeh et al^10^ reported that the Y58/E59 polymorphism increased the risk of GC development by 4.6 times. In terms of frequency, our data for the D58/K59 polymorphism were consistent with those of Shukla.^9^

The D58/K59/I60 polymorphism was found to be significantly associated with GC cases in our study (OR:58). Again, these combinations lost their significance in multivariate analysis. Gorrell et al^26^ reported that 58 and/or 59 amino acid polymorphisms were found together with the K62 or Q62 polymorphisms, and the authors concluded that this might be related to gastrointestinal pathologies. Our data on D58/K59/Q62 polymorphisms detected in the GC cases are consistent with Gorrell et al’s^26^ conclusions. Gorrell et al^26^ reported the polymorphisms in the 58 and 59 amino acids with neighboring rows, for example, with the Q62 or the E59 or N58 polymorphisms could be associated with GC.

In parallel with the possible hypothesis of Yeh et al^10^ we predict that atrophy with hypochlorhydria and the development of GC may be triggered in gastroduodenal pathologies related to *H. pylori* infections, including the D58/K59 polymorphism. In our study, the most common DKIGQ polymorphism was detected in 10 (52.6%) GC cases (OR: 58.5). Similar results were found also in our GC + DU cases. However, the D58/K59/I60/G61/Q62 combination lost its significance in multivariate analysis. In the study of Yadegar et al,^8^ NEIGQ and NKIGQ polymorphisms were 66.6% and 33.3% in GC cases, respectively, whereas in PUD cases, a different polymorphism, NKMGK, was detected in three (42.8%) cases.

Yadegar et al^8^ reported that the NEIGQ polymorphism is more common in GC cases. In the review of Gorrell et al.^26^ the most common polymorphisms at the international level are NEIGQ, NKIGQ, DKMGE, and DKIGK. Also, in this report, the prevalence of DKMGE in Africa as a geographic region suggests that it is an ancestral sequence for the motif in this region; although it differs slightly in the United States, the same motif is often seen, while European origins predominantly have NEIGQ and NKIGQ motifs. However, the same researchers pointed out that there is a different pattern for each region in Asia and that DKMGE is rarely seen, that this CagLHM region is very variable, and polymorphisms of the CagLHM region are specially formed from 58 to 59 two amino acid polymorphisms.

The amino acid polymorphic sequence diversity of the CagLHM region suggests that CagL has a versatile pathogenic role. The detection of polymorphisms in the CagLHM region is very important due to different evolutionary selections with the association of CagL with immune response. D58/K59 and DKIGQ were the most common polymorphisms in our cases with GC and are associated with the *vacA* genotype s1/m2.

It has been suggested that polymorphisms only in the CagLHM region may not be sufficient for GC development by *H. pylori*. Tegtmeyer et al^11^ and Tafreshi et al^12^ suggested that polymorphisms in another region of CagL may also have a role. Indeed, we detected the I134 polymorphism in 7 and 3 GC and DU cases, respectively. Yadegar et al^8^ detected the I134 polymorphism only in 1 of 3 cases with GC.

As the limitation of this study, we may indicate that CagA/EPIYA repeats that function integrated with this *cagL* region of *H. pylori *(actually, this study was based on CagA positivity, all cases were in *cagL*/*cagA* genopositive pattern) and genotypes of all *H. pylori *strains (such as BabA, VacAS1M1, or VacAS1M2) were not taken into the consideration. If these findings were taken into consideration, a more holistic, clearer, and inclusive approach could have been obtained. In this sense, since both CagA/EPIYA and different VacA genotypes and BabA results of all cases are in our records, a new study was planned based on these data.

As a result, the D58/K59 polymorphism had a significantly independent risk coefficient in GC (OR: 8.9) and GC + DU (OR: 6.5) cases. It was estimated that the risk of GC and DU development is 8.9 and 6.5 times higher with *H. pylori* strains carrying this polymorphism, respectively. The role of *CagL* in gastric carcinogenesis can be better revealed through different case-control-based studies and the identification of the D58/K59 polymorphism as a biomarker can both facilitate the identification of *H. pylori* (+) cases with high GC risk and contribute to the development of new therapeutics.

## Figures and Tables

**Table 1.** All Primer Sets Used in the PCR Analyses

Step	Primer Name	Sequence	Reference
Amplification of *cagA* gene	cagA-Forward	GAT AAC AGG CAA GCT TTT GAG G CTG	21
	cagA-Reverse	CAA AAG ATT GTT TGG CAG A	
Amplification of *cagL* gene	cagL-Forward	AGC CAA TTT TGA AGC GAA TG	10
	cagL-Reverse	CAA GCG TCT GTG GAA GCA GTG	
	cagL-sense	GAA GAT ATA ACA AGC GGT TT	7
	cagL- anti sense	TTT AAC AAT GAT CTT ACT TGA	
	cagL-Fwd-2	ACV AAG AGA CCA ACC ARC AAG	
	cagL-16	TCG CTT CAA AAT TGG CTT TC	
Amplification of *VacA* gene s1/s2 region	Forward	ATG GAA ATA CAA CAA ACA CAC	20
	Reverse	CTG CTT GAA TGC GCC AAA C	
Amplification of *VacA* gene m1/m2 region	Forward	CAA TCT GTC CAA TCA AGC GAG	20
	Reverse	GCG TCT AAA TAA TTC CAA GG	

**Table 2.** The Baseline Characteristics and the Cancer Type of the Cases

	Gastric Cancer	Duodenal Ulcer	Non-Ulcer Dyspepsia
Patients	19	16	26
Mean age (minimum–maximum)	57.316 ± 12.93 (31–79)	42.5 ± 10.97 (19–63)	51.77 ± 15.93 (22–76)
Gender (male/female)	14 (73.7%) 5 (26.3%)	7 (43.75%) 9 (56.25%)	12 (46.15%) 14 (53.85%)
Site of involvement	Intestinal type 17 (89.47%) Diffus type 2 (11.53%)	–	–
cagA (+) (n/%)	19 (100%)	16 (100%)	26 (100%)
cagL (+) (n/%)	19 (100%)	16 (100%)	26 (100%)

**Table 3.** The Comparison of the Study and Control Groups in Terms of Single Amino Acid Polymorphisms Detected in the cagLHM Region

Single Amino Acid Polymorphism	GC (n = 19)	NUD (n = 26)	*χ^2^*	*P*	OR	95% CI Min–Max
D58	16	4	21.061	.0001	29.333	5.750-149.625
K59	13	12	2.204	.1418	2.5278	0.734-8.709
I60	17	20	1.183	.435	2.550	0.454-14.326
S61	0	5	4.111	.1276	0.1002	0.052-1.9329
Q62	19	19	6.058	.0701	15.000	0.8003-281.1314
	DU (n = 16)	NUD (n = 26)	*χ^2^*	*P*	OR	95% CI Min–Max
D58	8	4	5.815	.032	5.500	1.293-23.389
K59	9	12	0.404	.751	1.500	0.428-5.251
I60	9	20	1.981	.187	0.386	0.101-1.480
S61	0	5	3.493	.1585	0.1185	0.061-2.2982
Q62	10	19	0.518	.510	0.614	0.162-2.327
	GC (n = 19)	DU (n = 16)	*χ^2^*	*P*	OR	95% CI Min-Max
D58	16	8	4.71	.029	5.33	1.1039-25.767
K59	13	9	0.55	.457	1.68	0.422-6.715
I60	17	9	5.01	.025	6.611	1.129-38.698
S61	0	0	–	–	–	–
Q62	19	10	8.37	.003	2414	1.2352-471.847
	GC + DU (n = 35)	NUD (n = 26)	χ^2^	*P*	OR	95% CI Min–Max
D58	24	4	16.994	.0001	12	3.329-43.259
K59	22	12	1.687	.150	1.974	0.704-5.540
I60	26	20	0.056	.867	0.867	0.285-2.838
S61	0	5	7.332	.0536	0.0551	0.029-1.0459
Q62	29	19	0.851	.271	1.781	0.518-6.119

GC, gastric cancer; DU, duodenal ulcer; NUD, non-ulcer dyspepsia; OR, odds ratio.

**Table 4.** The Comparison of the Study and Control Cases in Terms of Polymorphisms Containing the 58th and 59th Amino Acid Polymorphisms Detected in the cagLHM Region

Two Amino Acid Polymorphism	GC (n = 19)	NUD (n = 26)	*χ^2^*	*P*	OR	95% CI Min–Max
D58/K59	12	1	18.799	.0001	42.857	4.723-388.901
D58/E59	4	3	0.756	.433	2.044	0.400-10.457
N58/K59	1	11	7.704	.007	0.076	0.009-0.656
N58/E59	2	11	5.397	.024	0.160	0.031-0.843
	DU (n = 16)	NUD (n = 26)	*χ^2^*	*P*	OR	95% CI Min-Max
D58/K59	3	1	2.553	.146	5.769	0.545-61.121
D58/E59	5	3	2.496	.223	3.485	0.702-17.288
N58/K59	6	11	0.095	1.000	0.818	0.228-2.933
N58/E59	2	11	4.118	.084	0.195	0.037-1.038
	GC (n = 19)	DU (n = 16)	*χ^2^*	*P*	OR	95% CI Min–Max
D58/K59	12	3	6.99	.008	7.42	1.555-38.480
D58/E59	4	5	0.47	.491	0.586	0.127-2.703
N58/K59	1	6	5.64	.019	0.09	0.009-0.851
N58/E59	2	2	0.033	.854	0.82	0.102-6.6616
	GC + DU (n = 35)	NUD (n = 26)	*χ^2^*	*P*	OR	95% CI Min–Max
D58/K59	15	1	11.733	.001	18.750	2.278-154.332
D58/E59	9	3	1.897	.168	2.654	0.640-11.001
N58/K59	7	11	3.569	.059	0.341	0.109-1.062
N58/E59	4	11	7.671	.006	0.176	0.048-0.646

GC, gastric cancer; DU, duodenal Ulcer; NUD, non-ulcer dyspepsia; OR, odds ratio.

**Table 6.** Results of Logistic Regressions Analysis According to the Variables in Gastric Cancer Cases

Independent Variables	B	SE	Wald	Df	Sig	OR	95% CI (Min–Max)
Gender (Male)	0.436061	0.456673	0.911766	1	0.339646	1.546602	0.631914-3.785291
Age (younger than 55)	0.123748	0.463194	0.071376	1	0.789344	1.131731	0.456533-2.805527
D58/K59	2.192132	0.61517	12.69825	1	0.000366	8.954287	2.681623-29.89953
Constant	1.258691	0.659146	3.646485	1	0.056188	3.52081	

B, beta regression coefficient; SE, standard error; df, degree of freedom; OR, odds ratio; Sig, significance.

**Table 7.** Results of Logistic Regressions Analysis According to the Variables in Duodenal Ulcer Cases

Independent Variables	*B*	SE	Wald	Df	Sig	OR	95% CI (Min–Max)
Gender (Male)	−0.69182	0.454441	2.317557	1	0.127921	0.500665	0.205459-1.220022
Age (younger than 55)	−1.70789	0.638827	7.1475	1	0.007507	0.181248	0.051821-0.633932
Constant	1.010988	0.912207	1.228301	1	0.267738	2.748314	

B, beta regression coefficient; SE, standard error; df, degree of freedom; OR, odds ratio; Sig, significance.

**Table 8.** Results of Logistic Regressions Analysis According to the Variables in Patient Group (Gastric Cancer and Duodenal Ulcer Cases)

Independent Variables	*B*	SE	Wald	Df	Sig	OR	95% CI (Min–Max)
Gender (Male)	−0.15327	0.336195	0.20784	1	0.648465	0.857898	0.443881-1.65808
Age (younger than 55)	−0.58033	0.341955	2.880099	1	0.089681	0.559716	0.286348-1.094057
D58/K59	1.883836	0.600764	9.832842	1	0.001714	6.578695	2.026604-21.3555
Constant	2.039398	0.652601	9.765802	1	0.001778	7.685981	

B, beta regression coefficient; SE, standard error; df, degree of freedom; OR, odds ratio; Sig, significance.

**Table 5.** Association between CagL Polymorphisms and vacA Genotypes *H. pylori* DNA Isolates in GC and DU Cases

**cagL (n = 19)**	**vacA Genotypes in GC Patients**				***P***
	s1m1	s1m2	s2m1	s2m2	
D58/E59 (4)	0	2	0	2	
D58/K59 (12)	4	7	1	0	.022
N58/E59 (2)	1	0	0	1	
N58/K59 (1)	0	1	0	0	
DKIGQ (10)	3	6	1	0	.008
DEIGQ (4)	0	2	0	2	
**cagL (n = 16)**	**vacA Genotypes in DU Patients**				***P***
	s1m1	s1m2	s2m1	s2m2	
D58/E59 (5)	1	3	0	1	.59
D58/K59 (3)	0	2	1	0	
N58/E59 (2)	0	1	0	1	
N58/K59 (6)	4	2	0	0	.65
DKIGQ (1)	0	1	0	0	
DEIGQ (1)	1	0	0	0	

**Supplementary Table 1.** Polymorphisms Other Than the Polymorphisms of CagLHM Region

Polymorphism	Gastric Cancer (n = 19)	Duodenal Ulcer (n = 16)	Non-Ulcer Dyspepsia (n = 26)
T41	1 (5.26%)	–	2 (7.69%)
F48	–	–	1 (3.85%)
T56	–	–	8 (30.77%)
T72	2 (10.52%)	–	–
A112	1 (5.26%)	2 (12.5%)	1 (3.85%)
L118	–	1 (6.25%)	3 (11.54%)
K122	5 (26.3%)	2 (12.5%)	7 (26.92%)
I134	7 (36.8%)	3 (18.75%)	4 (15.38%)
G140	1 (5.26%)	2 (12.5%)	3 (11.54%)
A170	–	–	1 (3.85%)
V171	–	1 (6.25%)	1 (3.85%)
I175	2 (10.52%)	1 (6.25%)	–
K194	–	1 (6.25%)	5 (19.23%)
Q200	–	–	1 (3.85%)
I203	1 (5.26%)	1 (6.25%)	1 (3.85%)
K210	–	–	2 (7.69%)
S216	–	1 (6.25%)	1 (3.85%)
N221	–	2 (12.5%)	1 (3.85%)
